# Spin–orbit torque flash analog-to-digital converter

**DOI:** 10.1038/s41598-023-35845-7

**Published:** 2023-06-09

**Authors:** Hamdam Ghanatian, Luana Benetti, Pedro Anacleto, Tim Böhnert, Hooman Farkhani, Ricardo Ferreira, Farshad Moradi

**Affiliations:** 1 Department of Electrical and Computer Engineering, Aarhus University, 8200 Aarhus, Denmark; 2 International Iberian Nanotechnology Laboratory (INL), 4715-330 Braga, Portugal

**Keywords:** Electrical and electronic engineering, Electronic and spintronic devices, Magnetic devices, Electronic devices

## Abstract

Although analog-to-digital converters (ADCs) are critical components in mixed-signal integrated circuits (IC), their performance has not been improved significantly over the last decade. To achieve a radical improvement (compact, low power and reliable ADCs), spintronics can be considered as a proper candidate due to its compatibility with CMOS and wide applications in storage, neuromorphic computing, and so on. In this paper, a proof-of-concept of a 3-bit spin-CMOS Flash ADC using in-plane-anisotropy magnetic tunnel junctions (i-MTJs) with spin–orbit torque (SOT) switching mechanism is designed, fabricated and characterized. In this ADC, each MTJ plays the role of a comparator whose threshold is set by the engineering of the heavy metal (HM) width. Such an approach can reduce the ADC footprint. Monte-Carlo simulations based on the experimental measurements show the process variations/mismatch limits the accuracy of the proposed ADC to 2 bits. Moreover, the maximum differential nonlinearity (DNL) and integral nonlinearity (INL) are 0.739 LSB (least significant bit) and 0.7319 LSB, respectively.

## Introduction

ADCs translate analog input to digital output, and play a crucial role in computational systems^1–4^. With emerging computing in memory (CiM) for implementation of deep neural networks (DNN), the need for compact and low-power ADCs is increasing^5–7^. The conventional ADCs suffer from technology scaling due to the large process variation and lower performance in scaled nodes. According to the recent published roadmap for ADC, the ADC performance shows no obvious improvement in terms of resolution, area, and power consumption in the next few years using the current technology^8^. One promising solution can be shifting from conventional complementary metal–oxide–semiconductor (CMOS) technology to new hybrid technologies such as spin-CMOS technology^9^.

Magnetic tunnel junction (MTJ) is a promising candidate as a spintronic device for many applications due to its compatibility with CMOS, non-volatility, high retention time and long endurance^10–12^. An MTJ consists of an oxide layer sandwiched between two ferromagnetic (FM) layers. The magnetization direction of one of the FMs is fixed and it is called pinned layer (PL) while the other one that can be switched along its easy axis is called the free layer (FL). If the magnetization directions of the FL and PL are parallel, the device is in parallel state (P-state), where the MTJ presents a low resistance (logic ‘0’), whereas, if the magnetization direction of the FL is in the opposite direction of the PL, the device is in antiparallel state (AP-state) and shows a high resistance (logic ‘1’). The magnetic orientation of the FL can be adjusted by passing a charge current (*I*_STT_) through the MTJ via spin-transfer torque (STT) mechanism^13^. However, one of the challenges with this method for switching is that the thin oxide layer can be broken when the device experiences a high amount of *I*_STT_ leading to the reduction of reliability and endurance of MTJs^14^. Spin–orbit torque (SOT)-based MTJs have been proposed to overcome this issue while improving the switching efficiency^15^. In SOTs, a charge current (*I*_SOT_) greater than the critical charge current (*I*_SOT,crit_) flows through a heavy metal (HM) and the switching is accomplished by SOT through the spin Hall effect (SHE)^16,17^.

Recently, several works on designing ADC using SOT-based MTJ have been reported^8,18–21^. Jiang et al.^8^ have developed a spintronic ADC based on SHE and voltage-controlled magnetic anisotropy (VCMA). To tune *I*_SOT,crit_ of each MTJ, a resistive ladder is utilized to provide different voltages on the MTJs. Such an approach suffers from power overhead and reliability issues^18^. In other works^18–21^, a taper HM is shared between MTJs in which the width of the HM (w_HM_) is engineered to tune *I*_SOT,crit_. To sense the state of each MTJ in such approaches, a current flows through the MTJ (*I*_Sens_). However, considering the fact that the shared HM forms the bottom contact of the MTJs, *I*_Sens_ will pass through only a part of the HM. MTJs will experience different bottom contact resistance depending on their position on the shared HM. It is worth noting that different HM widths, obviously, lead to different HMs resistances in the path and this resistance gets larger for MTJs placed far from the HM terminal connected to the ground. The larger the resistance of the HM in the current path leads the larger the magnetoresistance (MR) degradation and thereby lower reading reliability. To overcome this issue, some works use a side-reading approach^18,19^, while other work uses a dummy quantizer to sense each MTJ resistance^20^. The difference in resistances of the adjacent HMs is compensated by adjusting the size of the transistor in the sensing circuit^21^. However, in the proposed solutions, increasing the complexity of the sensing circuit is the cost of mitigation issue of MR degradation. In this paper, the proof-of-the-concept of implementing an ADC based on spintronic devices is investigated which provides design guidelines for future spin-CMOS ADCs. To this end, a spin-CMOS ADC is proposed, designed, and characterized in which SOT-based MTJ and its *I*_SOT,crit_ act as a comparator and reference current (*I*_ref_) in conventional current-mode Flash ADCs, respectively. In spite of the proposed structures in literature^18–20^, in this structure, in-plane anisotropy SOT-based MTJs (i-SOT-MTJ)s are placed in parallel branches to mitigate the MR deduction and the complexity of the sensing circuit. The impact of the HM resistance on the MR is shown by comparing the measurement data extracted from the structure proposed by Ghanatian et al.^20^ with the approach presented in this paper. To compare the MR values between the two approaches, i-SOT-MTJ is employed. However, Ghanatian et al.^20^, used perpendicular anisotropy SOT-based MTJ (p-SOT-MTJ)s, in which the easy axis direction of the magnetic layers (i.e. FL and PL) is perpendicular to the plane of the magnetic layers. Compared to i-SOT-MTJ, p-SOT-MTJ offers several advantages, including fast switching and scalability^22^. However, in p-SOT-MTJ, switching is not deterministic and there is a need for an external magnetic field that it leads to an increase in complexity and process variation sensitivity. To overcome this issue, several techniques such as voltage control magnetic anisotropy (VCMA)^23^, exchange bias (EB)^24^, and SOT assisted by STT^20^ have been proposed. From the fabrication point of view, p-SOT-MTJs stacks are usually composed of ultrathin Co/Pt multilayers. This requires two additional targets in the deposition systems. Furthermore, in the inverted MTJ structure proposed (see Methods section), the reference layers are on top of the MTJ. The roughness caused by the lower layers is high and it is difficult to guarantee the perpendicular magnetic anisotropy (PMA) properties. Considering the nanofabrication challenges, we decided to use a stack where the FL is tilted slightly out-of-plane as described by Tarequzzaman et al.^25^. The measurement results show that the MR values of the proposed ADC are more than those of the structure proposed by Ghanatian et al.^20^ which means the reading reliability can be improved in the proposed structure.

In the approach proposed in this paper, the input current (*I*_in_) is copied to each branch and in case *I*_in_ is higher than *I*_SOT,crit_, the MTJ will switch. Hence, *I*_SOT,crit_ of each MTJ can behave like *I*_ref_ in the current-mode CMOS Flash ADCs. All MTJs are set in the P-state and if *I*_in_ > *I*_SOT,crit_, the MTJ is switched to the AP-state. w_HM_ is tuned so that the *I*_SOT,crit_ of each MTJ is compatible with reference currents (*I*_ref_, 2*I*_ref_, 3*I*_ref_, …) of the current-mode CMOS Flash ADC. Furthermore, Monte-Carlo simulation is performed to analyze the impact of the process variations/mismatch of MTJs and transistors on the reference currents of ADC. To this end, a random variable with a Gaussian distribution for MTJ is considered. The mean and standard deviation (σ) of the variable are defined by the measurement data of MTJs. Moreover, the variations of the CMOS circuit (the current mirror of *I*_in_) has been included to extract the reference currents of the ADC.

## Spin-CMOS ADC

**Figure 1:**
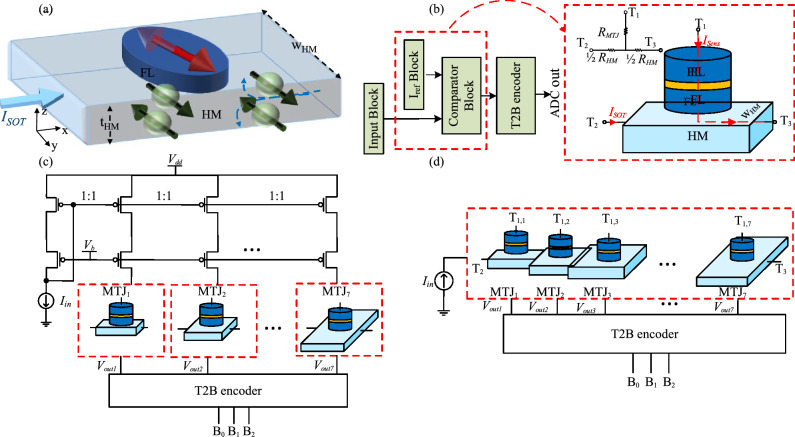
(**a**) The concept of SOT switching (**b**) The block diagram of the current-mode Flash ADC. The *I*_ref_ and comparator blocks can be replaced with SOT-based MTJ. (**c**) 3-bit spin-CMOS Flash ADC (parallel design) (**d**) 3-bit spin-CMOS Flash ADC (serial design).

The principle of the SOT switching mechanism in the FL of the SOT-based MTJ is shown in Fig. 1a. In this structure, a charge current (*I*_SOT_) flows through the HM along the x-direction. The SHE in the HM creates a pure spin current in z-direction, which is spin-polarized along the y-direction. This pure spin current generates an STT, which can switch the FL magnetization at a critical spin current density (*J*_SOT,crit_), which is similar for all MTJs that are nominally identical. The conversion efficiency between the charge current density and the spin current density is described by the spin Hall angle $\theta$. So, the *I*_SOT,crit_ can be described by^26–28^

1 $${I}_{\mathrm{SOT},\mathrm{crit}}={J}_{\mathrm{SOT},\mathrm{crit}}{\mathrm{t}}_{\mathrm{HM}}{\mathrm{w}}_{\mathrm{HM}}=\frac{2e}{\hslash }\frac{{J}_{\mathrm{s},\mathrm{crit}}}{\theta }{\mathrm{t}}_{\mathrm{HM}}{\mathrm{w}}_{\mathrm{HM}}$$

with the critical change current density (*J*_SOT,crit_)$,$ the electrons charge e, the electrons spin expressed by the reduced Planck’s constant $\frac{\mathrm{\hslash }}{2}$ and the HM thickness t_HM_. Thus, the charge current required for switching is proportional to w_HM_, which makes tuning of the critical charge currents relatively easy in these devices.

The schematic of the current-mode Flash ADC which consists of the input, *I*_ref_, comparator, and thermometer code to binary (T2B) encoder blocks are depicted in Fig. 1b. Flash ADCs are categorized into two groups: (1) voltage mode and (2) current mode. Current-mode Flash ADCs have some advantages over voltage-mode ADCs, such as less power consumption and the ability to operate with smaller supply voltages^21^. The input block makes several copies from *I*_in_, then the comparator block compares these copies with reference currents coming from *I*_ref_ block. The outputs of the comparator block are encoded by the T2B encoder and binary data corresponding to the input signal is generated as the ADC output. Hence, in the n-bit current-mode CMOS Flash ADC, 2^n^ − 1 copies of *I*_ref_ with different weights (i.e., *I*_ref0_, 2*I*_ref0_, …, (2^n^ − 1)*I*_ref0_) and *I*_in_ are required. The main idea of the proposed work is to replace the current mirror circuits needed for generating different copies of *I*_ref_ as well as the comparator block by an MTJ as shown in Fig. 1b. Since *I*_ref_ values are multiplications of *I*_ref0_, the size of transistors in the current mirror circuit will progressively increase. By replacing *I*_ref_ and comparator blocks with an MTJ, space and mismatch issues can be mitigated. As shown in Fig. 1b, *I*_SOT_ as an input current (*I*_in_) flows through the HM from T_2_ to T_3_ and as mentioned before the SOT-based MTJ acts as a comparator; hence it compares the *I*_in_ with its *I*_SOT,crit_ (behaves as the *I*_ref_ block). To sense the MTJ resistance, a current (*I*_Sens_) passes through the MTJ and a part of the HM from T_1_ to (T_2_/T_3_). The 3-bit spin-CMOS Flash ADC in two different designs called parallel and serial designs are shown in Fig. 1c and d, respectively. In both, seven i-SOT-MTJs are utilized to create an ADC with 3 bits of resolution. By engineering the w_HM_, *I*_SOT,crit_s can be tuned so that by increasing w_HM_, the required current for switching the MTJ will increase^29^. To this end, w_HM_ of each MTJ should be properly designed to ensure that *I*_SOT,crit_s for MTJ_1_, MTJ_2_, …, MTJ_7_ are equal with *I*_SOT,crit_, 2*I*_SOT,crit_, 3*I*_SOT,crit_, …, and 7*I*_SOT,crit_, respectively. In the serial design^18–20^, MTJs are put in series through HMs. As shown in Fig. 1d, by using this design, the input block (shown in Fig. 1b) that consists of the *I*_in_ mirror branches can be removed. However, the HM resistance (depending on the MTJ position) degrades the MR and the reading reliability. For instance, if T_2_ (Fig. 1d) is connected to the ground, the sensed resistance by *I*_Sens_ from T_1,7_ to T_2_ according to the equivalent resistive network of the MTJ depicted in Fig. 1b is *R*_MTJ7_ + 1/2 *R*_HM7_ + *R*_HM6_ + ⋯ + *R*_HM1_. Therefore, the MR for MTJ_1_ is *R*_MTJ*7*_(AP) − *R*_MTJ*7*_(P))/(*R*_MTJ7_(P) + 1/2*R*_HM7_ + R_HM6_ + ⋯ + R_HM1_) where, R_MTJ_(AP) and R_MTJ_(P) are MTJ resistance when MTJ is in AP-state and P-state, respectively. Moreover, the different resistance seen from T_1_ of each MTJ leads to an increase in the complexity of the sensing circuit. To mitigate this issue, a parallel design, as shown in Fig. 1c, is proposed in this paper. In this structure, MTJs are detached and the HM resistance seen from T_1_ of each MTJ is almost equal if all MTJs are in the same states. However, *I*_in_ should be copied by current mirrors (the input block) and fed into each MTJ. In both designs, the result of the comparison between *I*_in_ and *I*_SOT,crit_ in each MTJ is presented as a voltage signal (*V*_outi_ (1 ≤ i ≤ 7)). The T2B encoder block creates a 3-bit digital output (B_0_, B_1_, B_2_) based on *V*_outi_. The detail of the circuit design for sensing MTJ states and T2B are presented in^21^.

## Results and discussion

**Figure 2:**
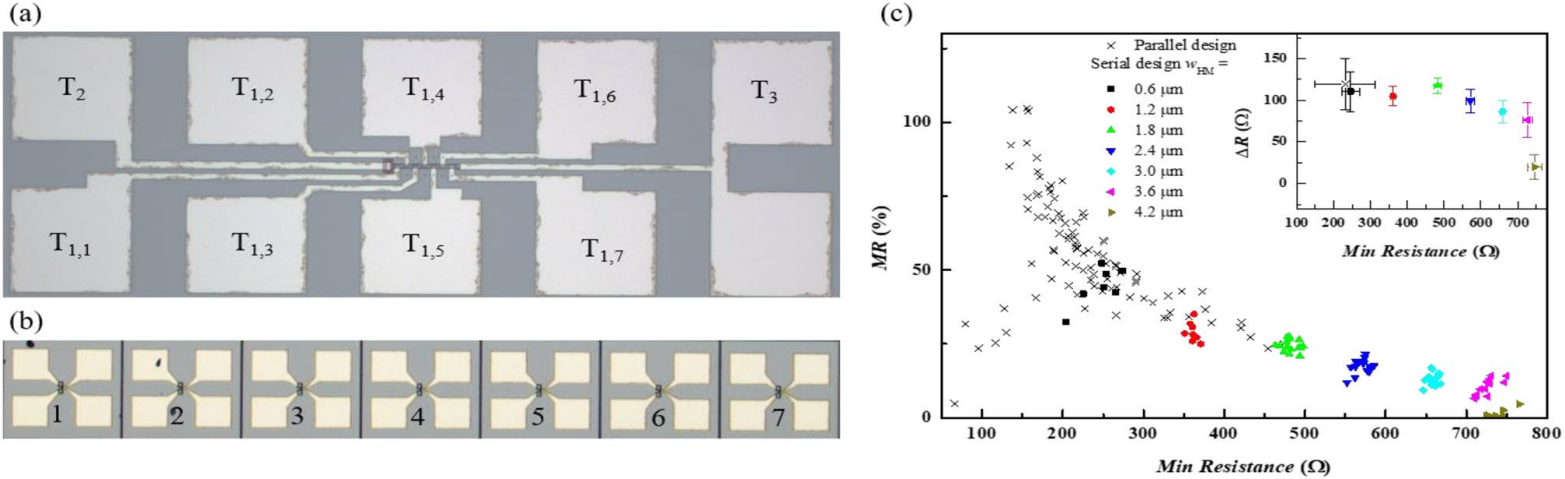
(**a**) Images from optical microscope of the serial design and (**b**) parallel design. (**c**) MR as function of the minimum resistance for serial and parallel designs for different w_HM_*,* inset the resistance variation.

The microscopic images of the serial and parallel designs are shown in Fig. 2a and b, respectively. Figure 2c shows the MR versus minimum resistance (the resistance seen by *I*_Sens_ when the MTJ is in the P-state) for the two designs. In the serial design, T_2_ is connected to the ground. MR dependency with the position of the MTJ is observed for the serial design in which the MR difference between the lowest (belongs to MTJ_7_) and highest (for MTJ_1_) is around 47%. The MR for the MTJs with a width of 4.2 µm is the lowest as compared to the other MTJs because as mentioned before, the resistance seen from T_1,7_ to T_2_ is larger. In general, MR in the serial design is lower than that in the parallel design because of the large HM resistance. Moreover, the dependency of MR to MTJ position is much smaller in the parallel design because the resistance seen from T_1_ of each MTJ to the ground is *R*_MTJ_ + *R*_HM_/2.

**Figure 3:**
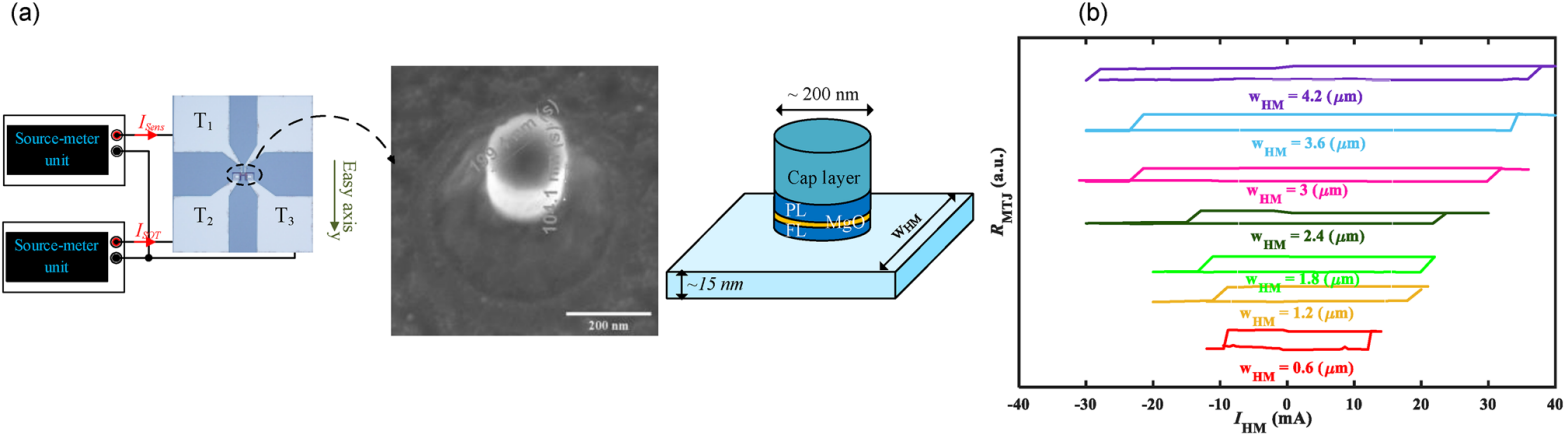
(**a**) The schematic view of the experimental setup used for characterization of the SOT-based MTJ (**b**) The R-I loop for different w_HM_.

The proof of concept of the implementation of a 3-bit Flash ADC based on the spintronic device can be investigated using the measured data from the characterization of the parallel configuration. To this end, the experimental setup of Fig. 3a is utilized to characterize the MTJs. All MTJs are initially set to the AP state by applying an external DC magnetic field with an amplitude of 19 mT along + y. Afterward, the external magnetic field is removed and *I*_*SOT*_ is injected into the HM through T_2_. Subsequently, *I*_*Sens*_ (a DC current) with an amplitude of 100 µA is applied by a source-meter unit to measure the resistance between T_1_ and T_3_. This resistance, according to the equivalent resistive network of MTJ (Fig. 1b) is *R*_MTJ_ + 1/2 *R*_HM_. In this measurement, the samples have been reported that the amount of change in their resistance after switching (*R*_MTJ_(AP) − *R*_MTJ_(P)) and their MR are more than 68 Ω and 20%, respectively. Figure 3b depicts the MTJ resistance versus *I*_SOT_ in the absence of the external magnetic field for 7 MTJs with different w_HM_. The positive (negative) current drives switching from P-state to AP-state (AP-state to P-state). In this paper, P-state is considered as the initial state of the MTJ 3-bit spin-CMOS Flash ADC and the switching from P-state to AP-state occurs (during the conversion phase in the ADC^20^) at the critical charge current called *I*_SOT,crit_ (P). During the reset phase in the ADC, MTJs are switched back to their initial states at the critical charge current called *I*_SOT,crit_ (AP), where the current direction is opposite of *I*_SOT,crit_ (P). Moreover, as shown in the obtained R-I loops, the width of the R-I loop becomes larger by increasing the w_HM_, which means that, as mentioned in Eq. (1), by increasing w_HM_, the *I*_SOT,crit_ (AP) and *I*_SOT,crit_ (P) are rising.

**Figure 4:**
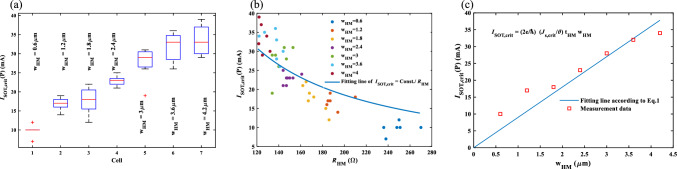
(**a**) The box plots of *I*_SOT,crit_ (P) for 7 cells with different w_HM_s (0.6 µm, 1.2 µm, 1.8 µm, 2.4 µm, 3 µm, 3.6 µm, 4.2 µm). (**b**) The distribution of *I*_SOT,crit_ (P) and *R*_HM_ for 7 cells (**c**) The average of *I*_SOT,ctit_ (P) for each cell versus the nominal value of w_HM_.

The box plots of *I*_SOT,crit_ (P) for seven cells are presented in Fig. 4a. w_HM_ of cell 1, 2, …, and 7 is 0.6 µm, 1.2 µm, …, and 4.2 µm, respectively. As shown in this figure, increasing w_HM_ results in an increasing trend in *I*_SOT,crit_ (P). σ of *I*_SOT,crit_ for cell 1, cell 2, … and cell 7 is 1.6 mA, 1.7 mA, 3.45 mA, 1.36 mA, 4.16 mA, 3.77 mA, 3.94 mA, respectively. The distribution of *I*_SOT,crit_ (P) and HM resistance (*R*_HM_), which are subdivided by seven cells *,* are depicted in Fig. 4b. The trend of increasing *I*_SOT,crit_ with *R*_HM_ according to the equation of *I*_SOT,crit_ (P) = const./*R*_HM_ [Eq. (1) and *R*_HM_ = const./(t_HM_ × w_HM_)] can be observed in this figure. Such large variations lead to nonlinearity, missing code and low accuracy issues in the ADC design based on the MTJs. The switching variation can be associated with the issue of domain wall dynamics^22^. However, in this experience, the lateral dimensions of the nanopillars are too small to show domain wall related effects. Such effects are more related to non-uniform magnetization structures such as vortex states, c-states or magnetization rotation to the out-of-plane direction^30,31^. In this work, a uniform in-plane magnetization can be expected as the free layer is very thin and the nanopillar diameter is quite wide (200 nm). Such random distributions are attributed to the variations in the w_HM_, t_HM_ and MTJs. In particular, t_HM_ is thin and the absolute variation is large that results in a large variation of the actual HM current density. Another way around, considering the nominal HM thickness this error results in a variation of the spin Hall angle. Reducing this variation is a technical challenge and it can be overcome by improving the nanopillar definition or by not using an inverted structure so that the SOT material is fabricated on top of the nanopillar.

*I*_SOT,crit_ (P) versus w_HM_ is presented in Fig. 4c in which the square points and the solid line are the measurement data and a fitting line, respectively. In this figure, each point is the average data of each cell that is extracted from Fig. 4a. The fitting line to the data with 0.8243 of R-squared (R^2^), represents a linear relation between *I*_SOT,crit_ and w_HM_ that is mentioned in Eq. (1). This linear dependency enables the linear ADC behavior. From the fitting line, we can determine the characteristic critical current density of the device *J*_SOT,crit_ = 0.6 × 10^12^ A m^−2^, which describes how efficiently the SOT current can switch the MTJs, which influences the precision of this ADC. Tarequzzaman et al.^26^ conducted a study on the critical current required to induce oscillations in similar MTJ nanopillars. However, it should be noted that in the mentioned study, the HM used was Tantalum. In that particular investigation, Tarequzzaman et al.^26^ obtained a critical current value for oscillations of *J*_SOT,crit_ = 0.33 × 10^12^ A m^−2^. It should be noted that a direct comparison between the current study, which focuses on the critical current for switching, and the previous study is not feasible due to the significantly larger critical current required for switching. Furthermore, Tungsten, the material employed in this current study, exhibits greater efficiency as a SOT material compared to Ta. However, despite these differences, a reasonable order of magnitude can still be inferred from this comparison in relation to the reference. It is worth considering that employing β–W phase may further reduce the critical current, which could be achieved through additional process engineering.

**Figure 5:**
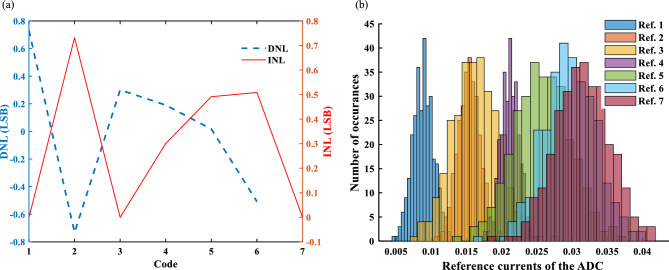
(**a**) DNL and INL of the 3-bit spin-CMOS flash ADC. (**b**) The distributions of the reference currents of ADC.

The differential nonlinearity (DNL) and integral nonlinearity (INL) characteristics for the proposed ADC are shown in Fig. 5a. The maximum DNL and INL are 0.739 LSB (5 mA) and 0.7319 LSB, respectively. The simulation results are obtained by a behavioral model for MTJs in Verilog-A that is extracted from the measurement. In this model, *I*_SOT,crit_ is the mean value of each cell that is extracted from Fig. 4c. The CMOS circuits (the current mirrors for *I*_in_) are simulated using Cadence in TSMC 180 nm technology. Monte–Carlo simulation is performed to evaluate the effects of the process variations/mismatch of the MTJs and CMOS circuits on the reference currents of ADC. The distributions of the reference currents shown in Fig. 5b are achieved by 300 simulation runs. Each plot includes the distributions of process variations and mismatch of the CMOS circuit of the *I*_in_ current mirror (Fig. 1c) and process variations of the related MTJ. For each MTJ, a behavioral model is considered that contains a variable with a Gaussian distribution. The values of mean and σ of the variable are extracted from Fig. 4a. ± 2σ yield can be supported only if MTJ_1_, MTJ_2_, MTJ_4_ and MTJ_7_ are employed while histograms of MTJ_3_, MTJ_5_ and MTJ_6_ strongly overlap with other reference current distributions. Therefore, according to Fig. 4b, the maximum available accuracy of the proposed ADC by such fabricated MTJs is 2 bits. The σ for first Ref.1, Ref.2, …, Ref.7 are 1.5 mA, 1.6 mA, 3.3 mA, 1.3 mA, 4 mA, 3.7 mA, 3.8 mA, respectively. The values of σ are almost the same ones extracted from Fig. 4a which means the process variation of MTJs is dominant as compared to the process variation and mismatch of the transistors.

## Conclusion

In this paper, i-SOT-MTJs are designed, fabricated, and characterized for the implementation of a 3-bit spin-CMOS Flash ADC. The linear relation between *I*_SOT,crit_ and the width of HM was verified and the figure of merit of the i-SOT-MTJ (*J*_SOT,crit_) is 0.6 × 10^12^ A m^−2^. Seven separated i-SOT-MTJs with different width of HMs are employed. In this structure, MTJ and its *I*_SOT,crit_ play the role of the comparators and *I*_ref_ blocks in Flash ADC, respectively. Hence, the power-hungry comparators and the current mirrors that generate *I*_refs_ in current-mode Flash CMOS ADCs are eliminated. The current used for sensing the MTJ resistance sense the HM resistance of only one MTJ in the path leading to significant improvement in MR and reading reliability. The maximum INL and DNL are in the range of 0.7319 LSB and 0.739 LSB, respectively. Furthermore, Monte-Carlo simulations are conducted for the estimation of the ADC accuracy in the presence of the process variation/mismatch of the MTJ and CMOS transistors. The simulation results show the accuracy of the proposed ADC limits to 2 bits, which can be enhanced by improving the MTJ fabrication process in the future.

## Methods

An inverted MTJ stack with a 3-terminal geometry, similar to those used in previous works^26,32,33^, was proposed. The MTJ consists in 15 W/ 1.4 CoFe_40_B_20_/MgO/2.2 CoFe_40_B_20_/0.85 Ru/2.5 CoFe_30_/6 IrMn/5 Ru/140 Cu/30 Ru (thicknesses in nanometer) deposited on Si (100)/200 nm thermal SiO_2_ by magnetron sputtering. The MgO thickness was targeted to have a resistance-area product (R × A) of 12 Ω µm^2^, as below 10 Ω µm^2^, a decrease in tunnel magnetoresistance (TMR) is observed^34^. Through current-in-plane transport measurements, the stack exhibited an R × A of 14.3 Ω µm^2^ and a TMR of 144%. Tungsten (W) in the stack was chosen as heavy metal due to its high spin hall angle reported in the β-phase^35^. However, this phase is only possible for W thicknesses of a few nanometers (< 6 nm)^36^ which is rather challenging for device fabrication since it reduces the stopping point margin for the pillar etch. By tuning the deposition conditions or incorporating some defects, it is possible to increase the thickness of the β–W^37,38^. As a compromise, we decided to use a 15 nm W layer. Thus, it is likely that this layer is in the α–W phase in the presented devices.

The nanofabrication process is the same one described by Tarequzzaman et al.^32^. Electron beam lithography (EBL) was used to pattern 200 nm diameter nanopillars and an ion beam milling system was used for etching. Through the secondary ion mass spectrometry incorporated into the etching system it was able to control the etch and stop within the 15 nm W layer. In order to ensure electrical isolation and physical stability, the nanopillars were buried into 800 nm SiO_2_ and planarized by ion beam milling with grazing incidence to expose the top of the pillar. The EBL was also used to define the HM line bottom electrode with a 6 µm length and width varying from 0.6 to 4.2 µm. Direct laser writing was used in the others lithographies in order to establish electrical contact with top and bottom electrodes.

After the nanofabrication, the devices were annealed at 300 °C for 2 h, with an applied magnetic field of 1 T along the same axis direction of the field used during the deposition in order to pin the synthetic antiferromagnetic layers. After the annealing the free layer of 1.4 nm CoFe_40_B_20_ exhibits in plane magnetic anisotropy^32^.

## Data Availability

The data that support the findings of this study are available from the corresponding author upon reasonable request.
